# Supplementary material for: Soft-interfaced liquid crystal microfluidics can probe the rigidity of lipid vesicles
Source: Commun Mater. 2026 Mar 17;7(1):120. doi: 10.1038/s43246-026-01128-7 (PMC13143827; doi:10.1038/s43246-026-01128-7)
Supplement: Supplementary file 2 — Supplementary Information [file 43246_2026_1128_MOESM2_ESM.pdf]

Supplementary Information

Soft-Interfaced Liquid Crystal Microfluidics Can Probe the Rigidity of Lipid Vesicles

Cansu Dedeoğlu and Emre Bukusoglu

Department of Chemical Engineering, Middle East Technical University, Dumlupınar Bulvarı No.1, Çankaya, 06800 Ankara, Türkiye.

Corresponding author: emrebuk@metu.edu.tr

Shear Stress and Shear Rate Calculations for Microfluidic Channels

Experimental values of water velocity were obtained via particle tracking in the channel, and data were averaged.

We calculated the volumetric flow rate using,

$$\dot{Q} = v * A \qquad (1)$$

where $\dot{Q}$ is volumetric flow rate (m^3/s), v is average experimental velocity (m/s), and A is the cross-sectional area of water flow side of the channel. The channel dimensions were such that h is the depth of the channel, 14 µm, and w is the width of the aqueous phase, 250 µm.

Maximum shear stress at the wall (parallel-plate approximation)[1];

$$\tau_w \simeq \frac{h}{2} * \frac{\Delta P_{flow}}{L} \qquad (2)$$

$$\tau_w = \frac{14 * 10^{-6} m}{2} * \frac{500\ Pa}{0.025\ m} = 0.14\ Pa$$

where τ_w is the maximum shear stress at the wall, ΔP_{flow} is the pressure drop through the channel, and L is the length of the channel. ΔP_{flow} assumed as set pressure since the end of the channel is open to the atmosphere.

Using Newton's law of viscosity,

$$\tau_w = \mu * \dot{\gamma} \qquad (3)$$

where μ is the viscosity of the PBS at room temperature and $\dot{\gamma}$ is shear rate, we calculate the shear rate in the microfluidic channels. The calculation results are shown in Table S1 for four different flow rates measured.

Table S1. The calculated values for shear stress and shear rate of the aqueous phase at given inlet pressures to the channels

	5 mbar	7.5 mbar	25 mbar	30 mbar
Water velocity (m/s)	$1.35*10^{-4}$	$2.62*10^{-4}$	$6.72*10^{-4}$	$7.89*10^{-4}$
Flowrate (m^3/s)	$4.73*10^{-13}$	$9.17*10^{-13}$	$2.35*10^{-13}$	$2.72*10^{-13}$
Shear stress (Pa)	0.14	0.21	0.70	0.84
Shear rate (s^{-1})	158	237	787	948

Shear Rate Calculations for Droplet Systems

To find the angular velocity of the vortex[2]

$$\omega = \frac{2\pi * \mathrm{RPM}}{60}$$

where ω is the angular velocity

$$\omega = \frac{2\pi * 3000}{60} = 314.2\ rad/s$$

To find the shear that applied the solution in 4 mL (d=1.2 cm),

$$\dot{\gamma} = \frac{\omega * R}{d}$$

$$\dot{\gamma} = \frac{314.2\ * 0.006}{0.001} \approx 1900\ s^{-1}$$

Supporting Figures

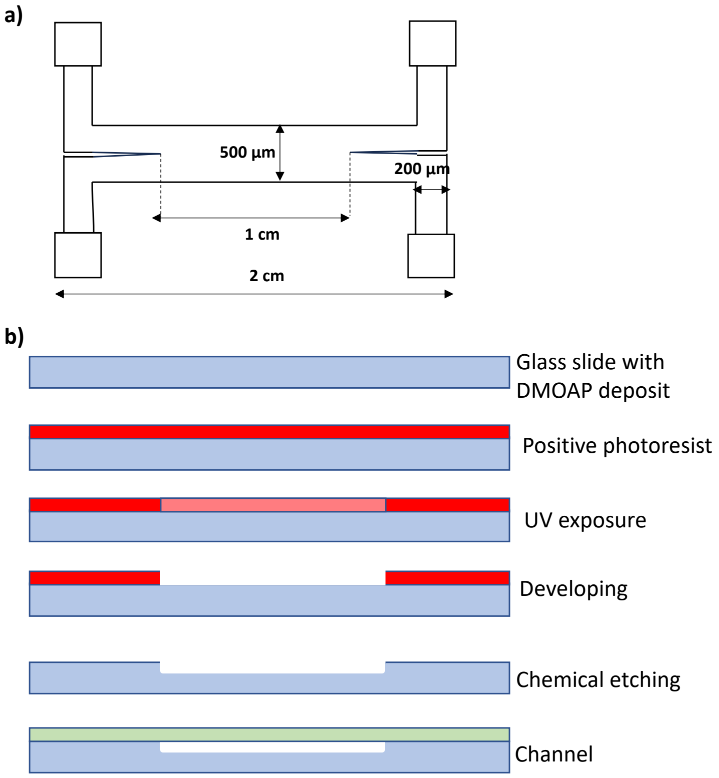

Figure S1. a) Dimensions of the mask used in the lithography process of the microfluidic channels, b) Sketches showing the major fabrication steps of the microfluidic channel.

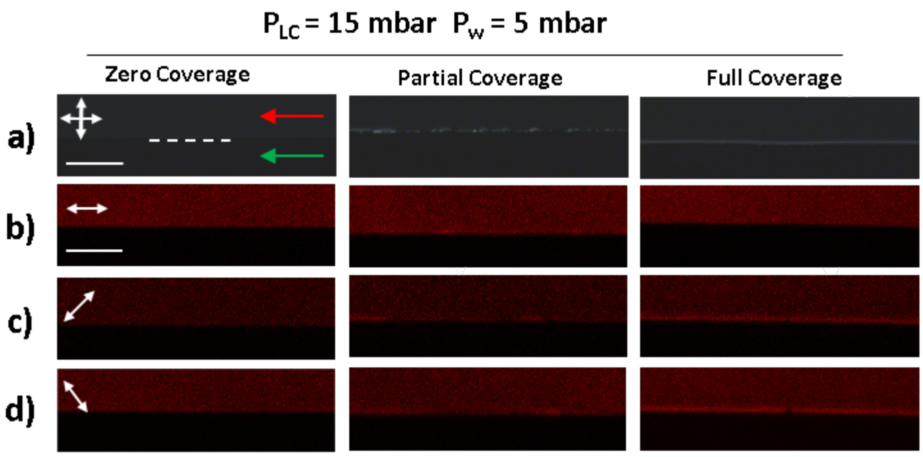

Figure S2. Images of the channels with and without DLPC adsorption. a) Polarized optical micrographs of the LC-aqueous microfluidic channel under 0° analyzer and 90° polarizer, FCPM images of the same channel at b) 0°, c) 45°, and d) 135° polarization of the excitation source. The dashed line in the images indicates the location of the horizontal soft interfaces. Scale bars: 100 µm.

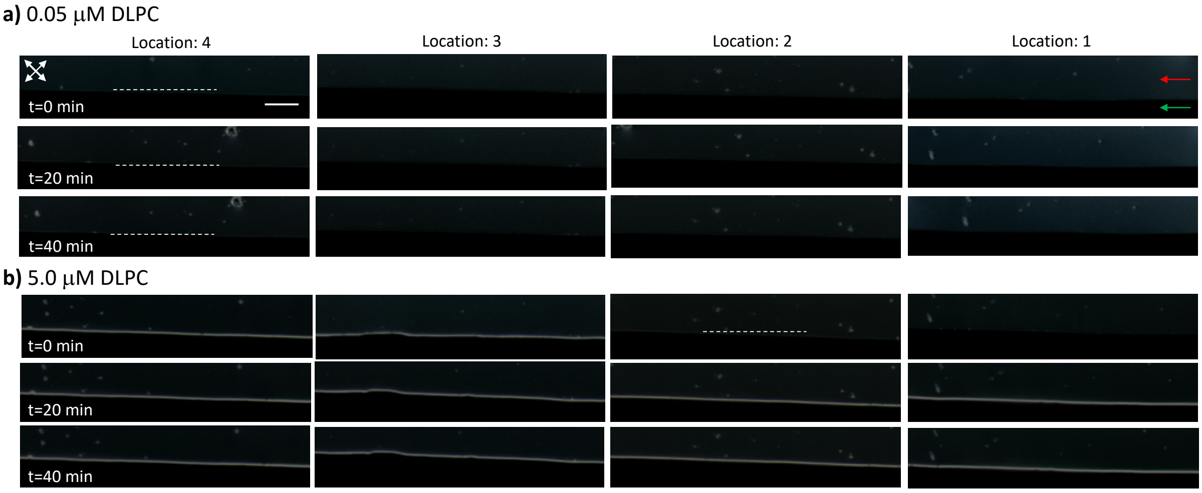

Figure S3. Polarized optical micrographs of the microfluidic LC-aqueous interface at locations 1-4 by formed using a) 0.05 μM and b) 5.0 μM concentrations of the DLPC feed. Images were collected under a 45° analyzer 135° polarizer. Images were collected at the duration after aqueous phase flow was initiated, as indicated in the images. The white double-sided arrow shows the orientations of the analyzer and polarizers used in all of the images shown in the figure. The dashed lines in the images indicate the location of the horizontal soft interfaces. Scale bar: 100 μm, common for all images. Scale bar: 100 μm.

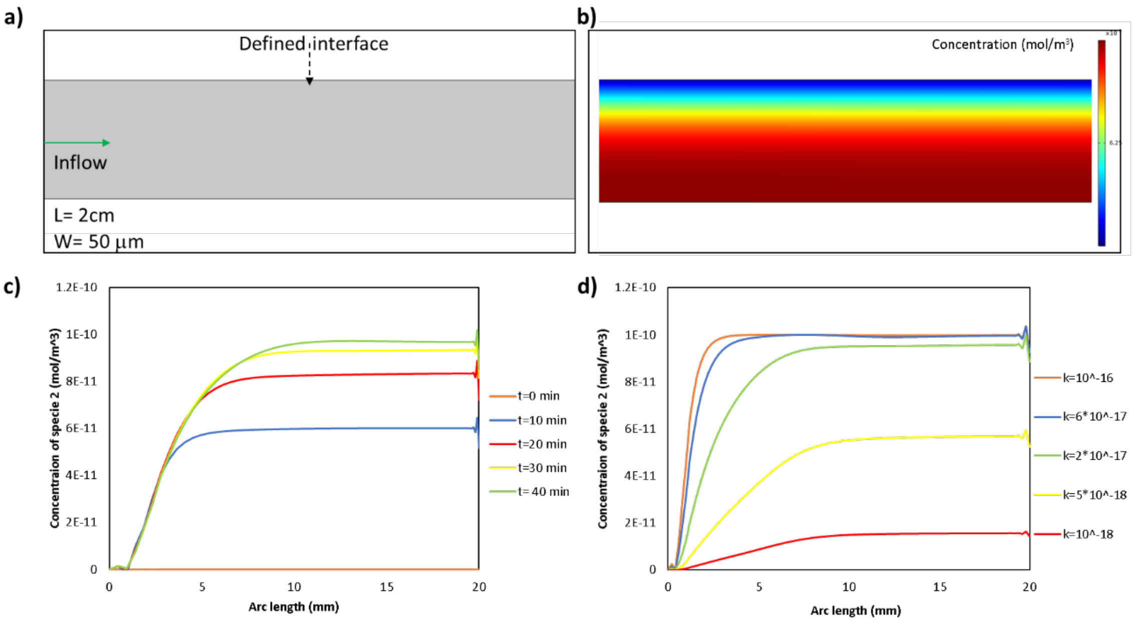

Figure S4. a) Geometry defined in COMSOL represents the water side of the experimental microfluidic design, b) the concentration profile of species 1, which defines the lipid vesicles in the bulk, c) concentration distribution of species 2, which defines the single lipid molecules along the interface length with respect to time, c) concentration distribution of species 2, which defines the single lipid molecules along the interface length with respect to different surface reaction constants.

Details of Fig. S4. By using COMSOL Multiphysics, we defined a rectangular geometry with dimensions of 50 µm width and 2 cm length to represent the aqueous side of our microfluidic channels (Fig. S4a). We defined the creeping flow module with a no-slip wall for the hard interface and a slip wall with 0.8 µm/s velocity for the interface. Also, for the inlet velocity, we defined 135 µm/s from our experiments. By using the transport of diluted species module of COMSOL and defining the adsorption, we used a surface reaction model with two species, where the first species consumes and represents lipid vesicles in the bulk, and the second species forms in the reaction model, representing the single lipids at the interface. Also, we limited the surface reaction with the Langmuir model.[3]

$$J_{c1} = (-k * c_1 * \left(1 - \frac{c_2}{c_{2,max}}\right)) \qquad (5)$$

$$J_{c2} = \left(80000 * k * c_1 * \left(1 - \frac{c_2}{c_{2,max}}\right)\right) - k_2 * c_2 \qquad (6)$$

Where J_{c1} and J_{c2} represent the mol flux of the first species and the second species, respectively. k and k_2 define the surface reaction rate constants. c_1 and c_2 represent the concentration of lipids in the bulk and at the interface, respectively, while $c_{2,max}$ was defined as the maximum concentration at the interface to correlate the monolayer coverage. In equation 2, the right-hand side terms define the desorption of the lipids from the interface, and desorption is represented by k_2. However, phospholipid adsorption into the interface is irreversible. Therefore, we defined k_2 as very small to avoid the desorption. Moreover, multiplying equation 2 by 80000 represents the single lipid number in a vesicle, since lipids are in vesicles at the bulk; however, after adsorption, they are located at the interface as single molecules, and it was calculated by considering the area per lipid is 0.5 $\frac{nm^2}{molecule}$ and radius of a vesicle is approximately 40 nm and assuming the bilayer thickness is negligible.

$$2 * (4 * \pi * (40\ nm)^2) = 40212.4\ nm^2$$

$$40.212.4\ nm^2 * \frac{1}{\frac{0.5\ nm^2}{lipid}} \simeq 80000\ lipid$$

The concentration profile of the first species through the channel (Fig. S4b) shows that c_1 decreases through the y direction or interface since surface reaction occurs at the interface, and c1 was defined as being consumed in this reaction. Also, c1 concentration decreases gradually through the end of the channel, region where c1 decreases increases. Concentration profile of the second species, lipids at the interface, analyzed with respect to time and arc length, which is a cut line at the interface (Fig. S4c). Profile showed that c2 increases with increasing arc length; this trend was consistent with the phospholipid domain accumulation at the end of the channel in our experiments. To relate change in the rigidity of the vesicles, we changed the surface reaction constants in the simulation. We found that monolayer coverage cannot be reached with small k's, meaning vesicles were not able to adsorb into the interface. With increasing k values monolayer coverage was reached faster, and single lipids located at the small arc lengths, representing adsorption, occurred at the longer region of the interface with increasing k values. This trend can be correlated with the soft vesicles, such as mixed micelles with DTAB or egg SM with cholesterol, and stiff vesicles, such as DLPC with C16 or pure egg SM, as kinetic in our experiments. High k values absorption kinetics represented the soft vesicles, while small k's represent the stiff vesicles' adsorption kinetics. Although the concentration profile of c2 with time and arc length was not exactly the same as our experiments since we did not define 5CB, and therefore nematic elasticity, the simulation gave insights about the adsorption kinetics and monitoring the rigidity effect.

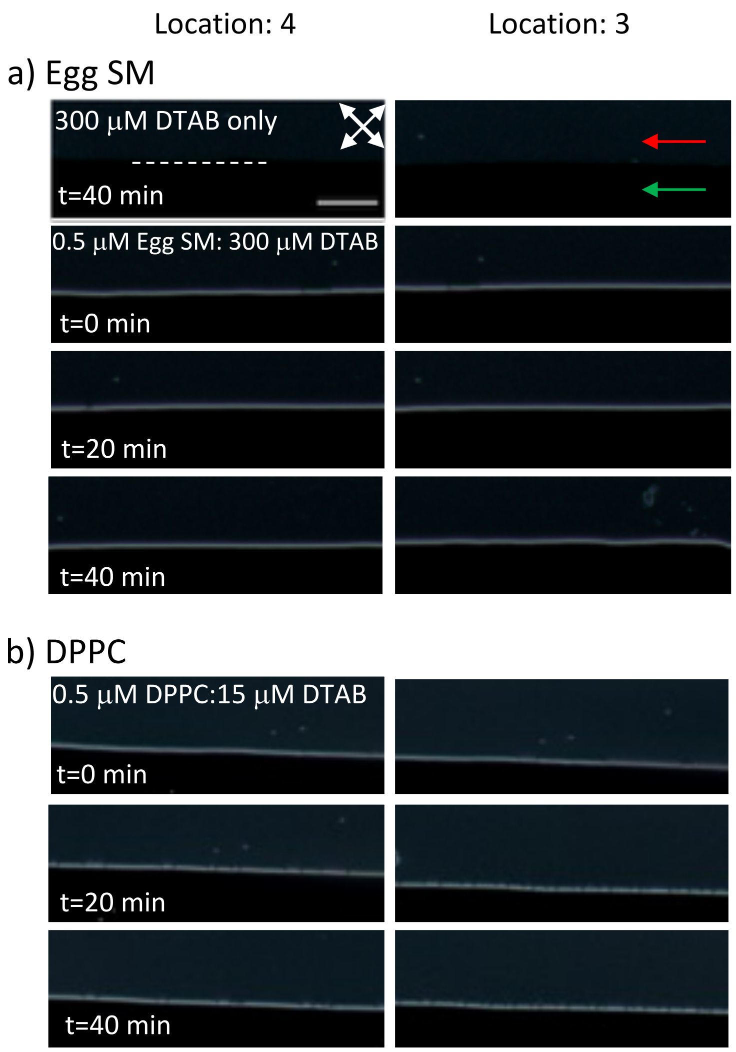

Figure S5. The polarized optical micrographs are shown that were taken from the 14 μm-deep microfluidic channels where the aqueous phases were composed of vesicles formed by (a) 0.5 μM Egg SM: 300 μM DTAB, and (b) 0.5 μM DPPC: 15 μM DTAB. Each line of images was collected from locations 4 and 3 as indicated in the top title. Images collected from experiments with 300 μM DTAB in the first line. Images collected from experiments 0.5 μM Egg SM: 300 μM DTAB and 0.5 μM DPPC: 15 μM DTAB were collected at the duration after aqueous phase flow was initiated, as indicated in the images. The white double-sided arrow shows the orientations of the analyzer and polarizers used in all of the images shown in the figure. The dashed lines in the images indicate the location of the horizontal soft interfaces. Scale bar: 100 μm, common for all images.

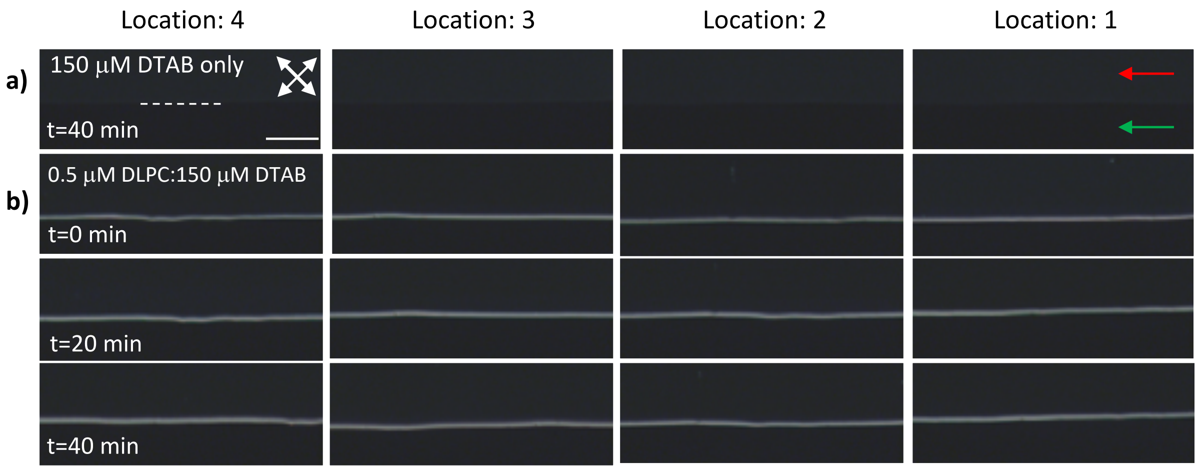

Figure S6. The polarized optical micrographs are shown that were taken from the 14 µm-deep microfluidic channels where the aqueous phases were composed of (a) 150 µM DTAB, and (b) 0.5 µM DLPC: 150 µM DTAB. Each line of images was collected from locations 1-4 as indicated in the top title. Images were collected at duration after aqueous phase flow was initiated, as indicated in the images. The white double-sided arrow shows the orientations of the analyzer and polarizers used in all of the images shown in the figure. The dashed lines in the images indicate the location of the horizontal soft interfaces. Scale bar: 100 µm, common for all images.

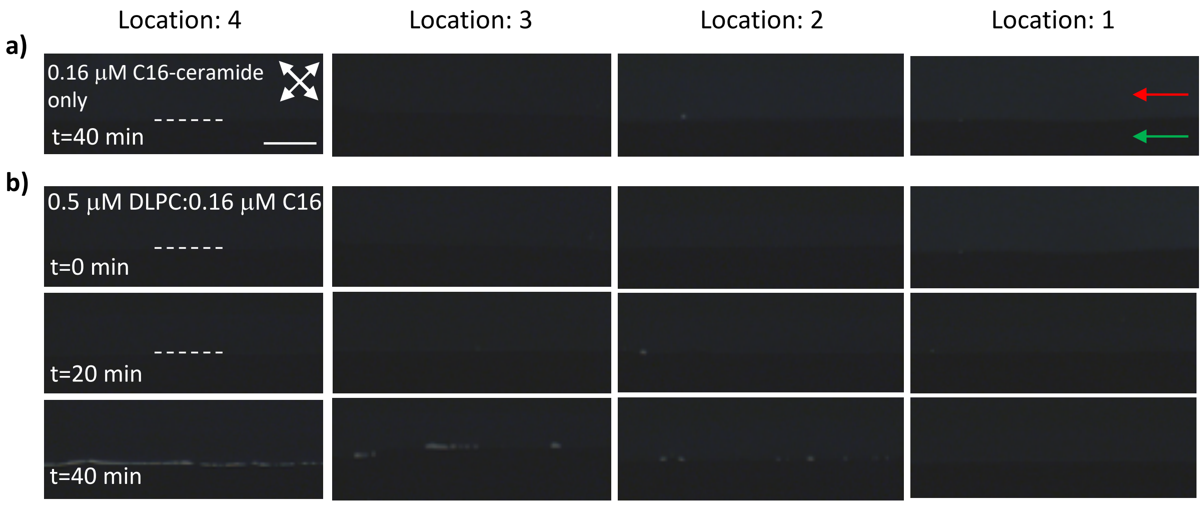

Figure S7. The polarized optical micrographs are shown that were taken from the 14 μm-deep microfluidic channels where the aqueous phases were composed of (a) 0.16 μM C16-ceramide, and (b) 0.5 μM DLPC:0.16 μM C16-ceramide. Each line of images was collected from locations 1-4 as indicated in the top title. Images were collected at duration after aqueous phase flow was initiated, as indicated in the images. The white double-sided arrow shows the orientations of the analyzer and polarizers used in all of the images shown in the figure. The dashed lines in the images indicate the location of the horizontal soft interfaces. Scale bar: 100 μm.

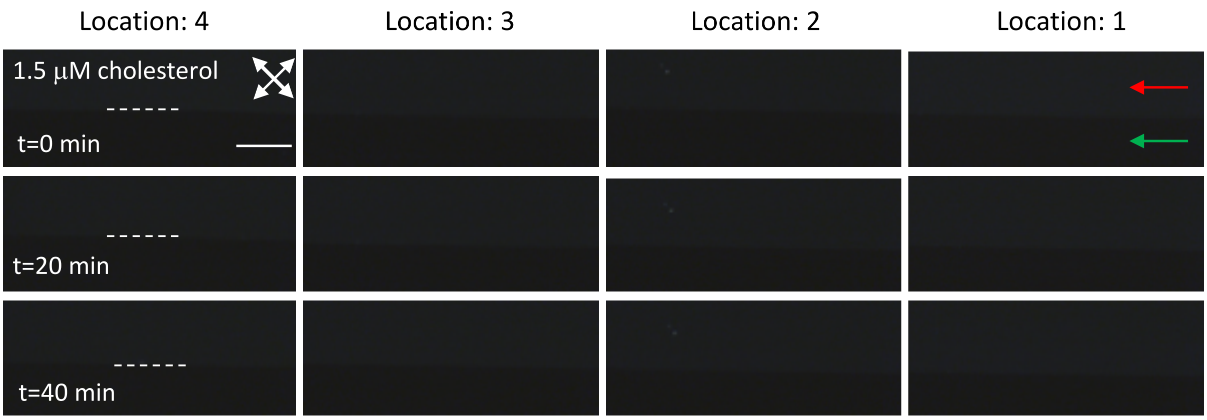

Figure S8. The polarized optical micrographs are shown that were taken from the 14 μm-deep microfluidic channels, where the aqueous phases were composed of 1.5 μM cholesterol. Each line of images was collected from locations 1-4 as indicated in the top title. Images were collected at the duration after aqueous phase flow was initiated, as indicated in the images. The white double-sided arrow shows the orientations of the analyzer and polarizers used in all of the images shown in the figure. The dashed lines in the images indicate the location of the horizontal soft interfaces. Scale bar: 100 μm.

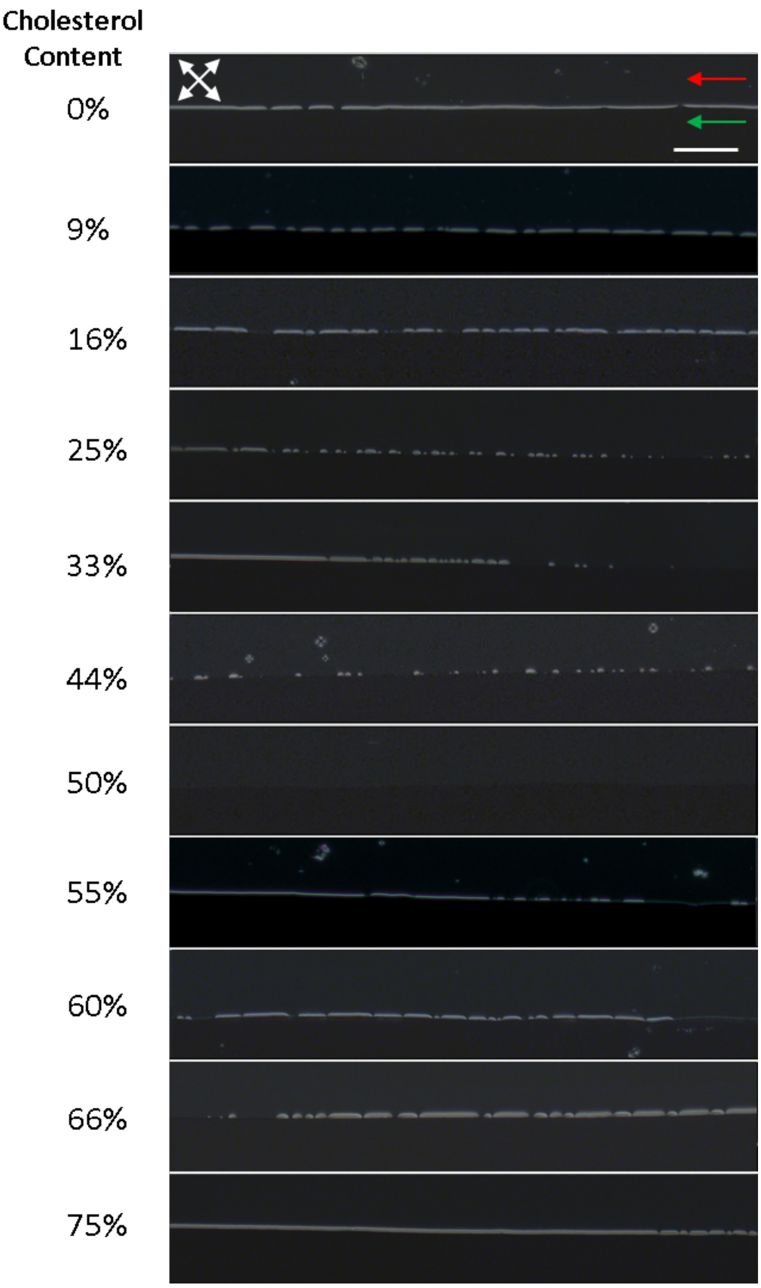

Figure S9. Polarized optical micrographs collected from experiments with contacting 5CB with a constant 0.5 μM DLPC concentration and varying content of cholesterol from 0% to 75%, as indicated in the near of images. Images were collected at location 4 and t=40 min. The white double-sided arrow shows the orientations of the analyzer and polarizers used in all of the images shown in the figure. Scale bar: 100 μm.

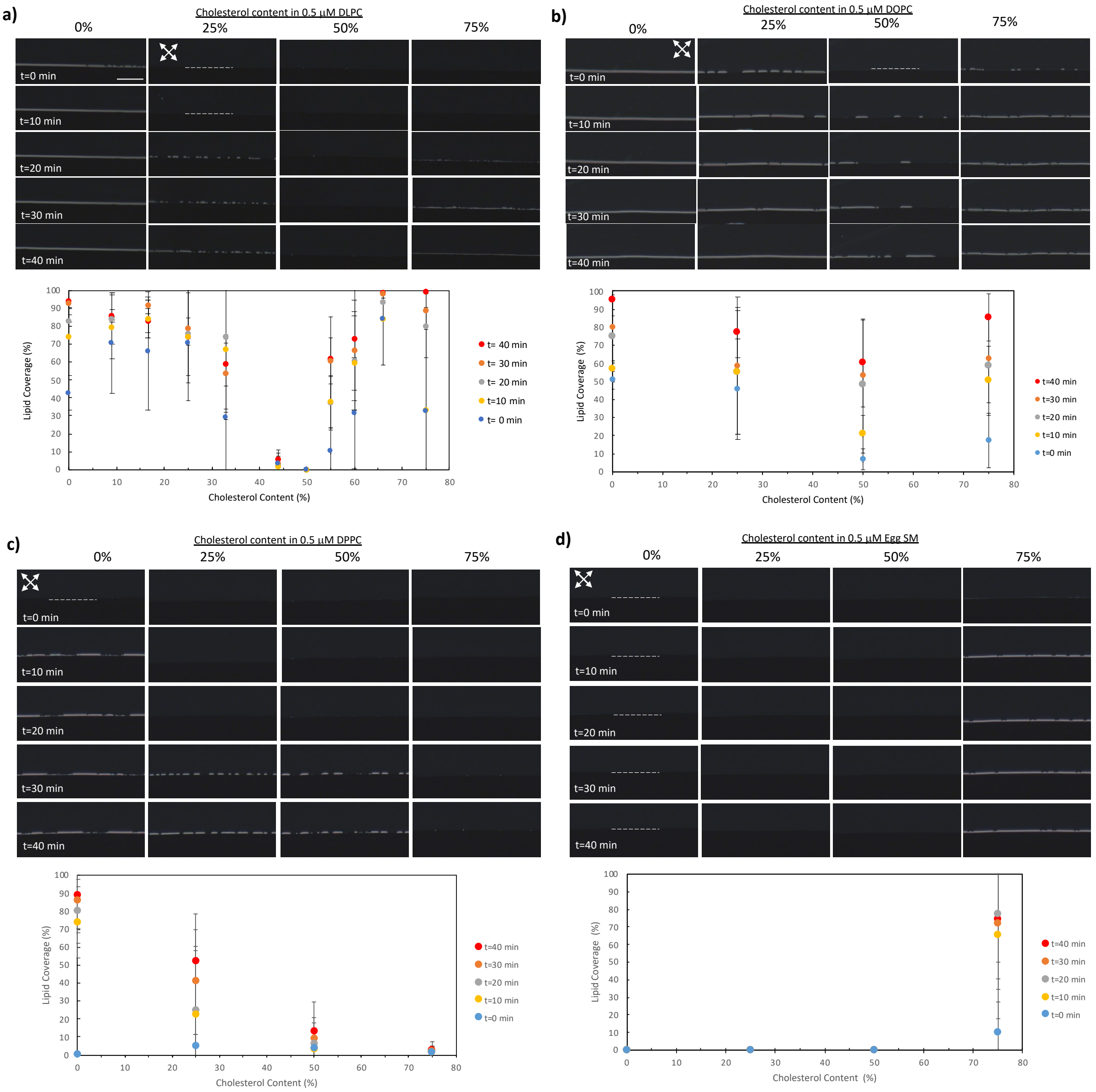

Figure S10. Polarized optical micrographs collected from experiments with contacting 5CB with a constant 0.5 μM (a) DLPC, (b) DOPC, (c) DPPC, (d) Egg SM, and varying content of cholesterol from 0% to 75%, as indicated in the top titles. Each line of images was collected at the duration after aqueous phase flow was initiated, as indicated in the images. All images were collected at t = 40 min. The white double-sided arrow shows the orientations of the analyzer and polarizers used in all of the images shown in the figure. The dashed lines in the images indicate the location of the horizontal soft interfaces. The plots of lipid coverage (%) vs cholesterol content (%) for each lipid were given under the micrographs.

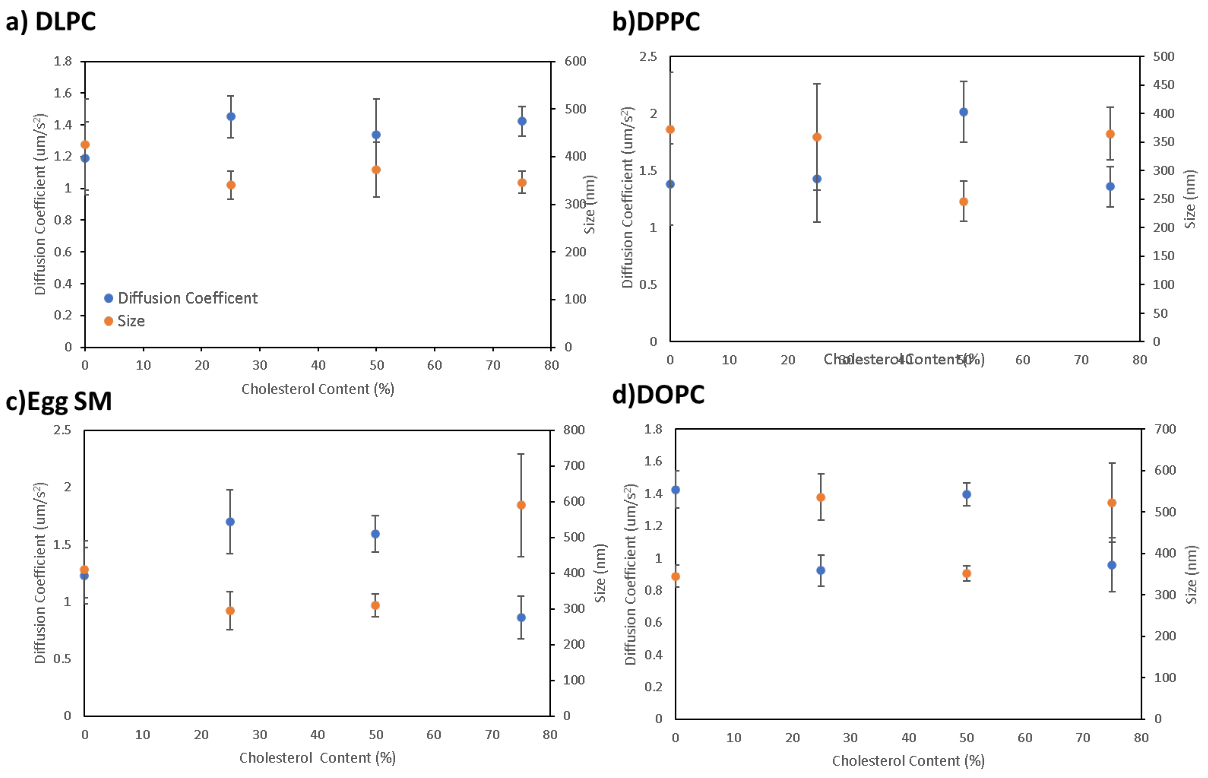

Figure S11. The plots of diffusion coefficients (left y-axis) and size (right y-axis) of the vesicles formed by (a) DLPC, (b) DPPC, (c) Egg SM, (d) DOPC with cholesterol sketched vs. cholesterol content (%).

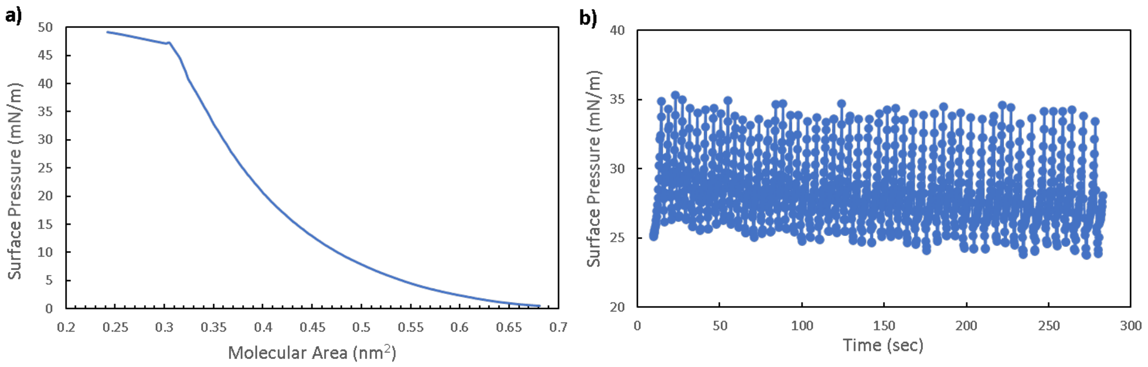

Figure S12. The plots of (a) the Langmuir isotherm and (b) the oscillating surface pressure with time for DLPC.

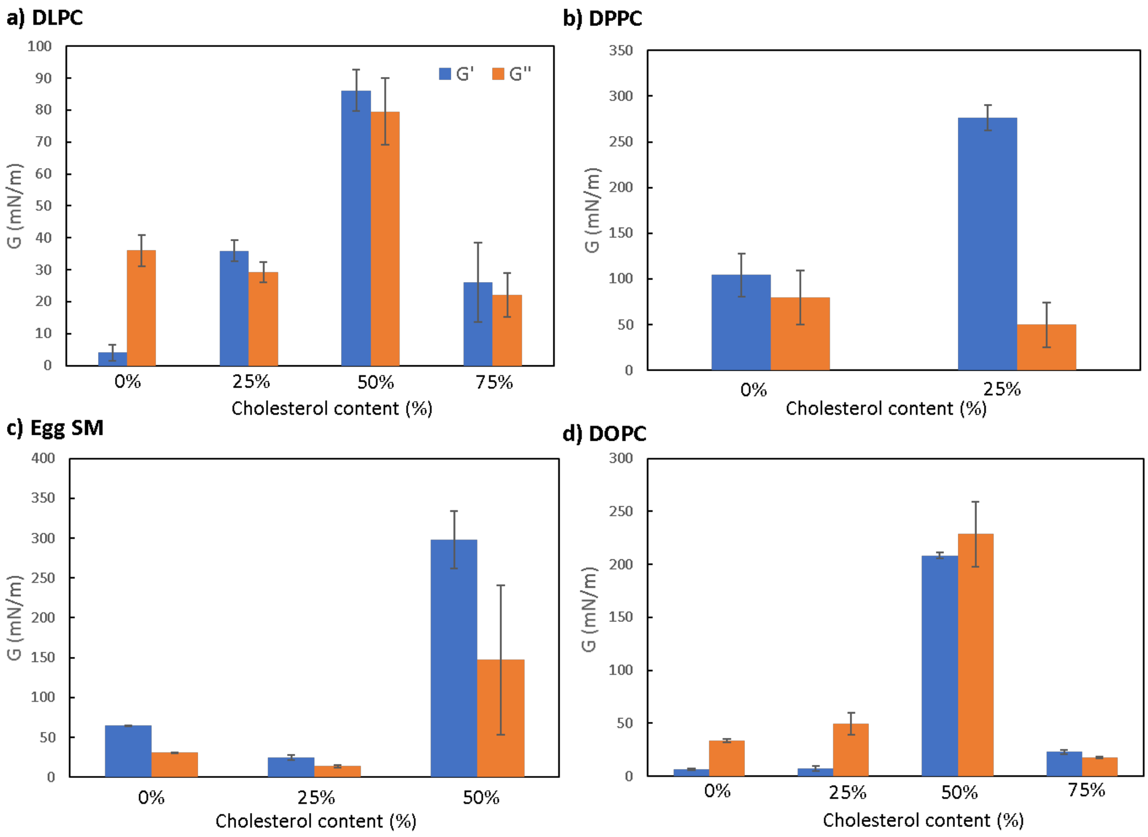

Figure S13. The plots of surface elastic modulus (G’) and surface loss modulus (G’’) with cholesterol content for (a) DLPC, (b) DPPC, (c) Egg SM, and (d) DOPC.

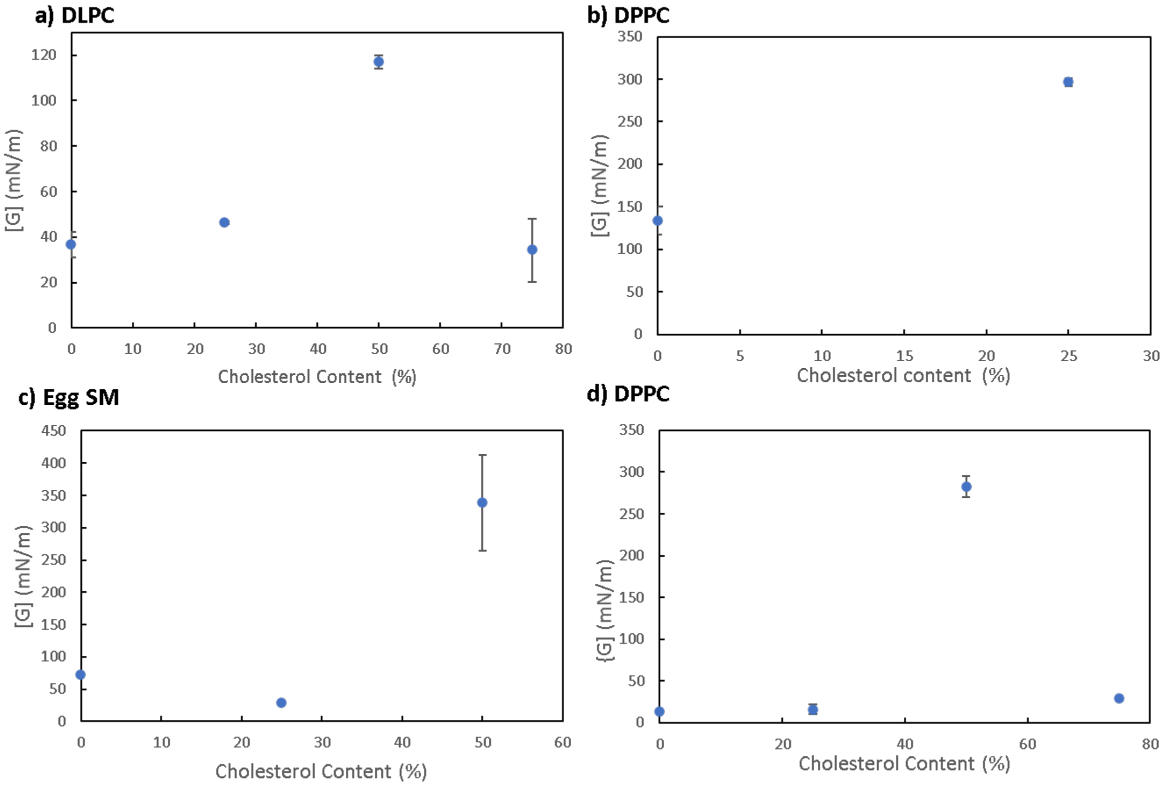

Figure S14. The plots of surface elasticity with cholesterol content and sample plots for oscillating barrier experiments for (a) DLPC and DLPC with 25% cholesterol, (b)DPPC and pure DPPC, (c) Egg SM and with 50% cholesterol, and (d) DOPC with 75% cholesterol.

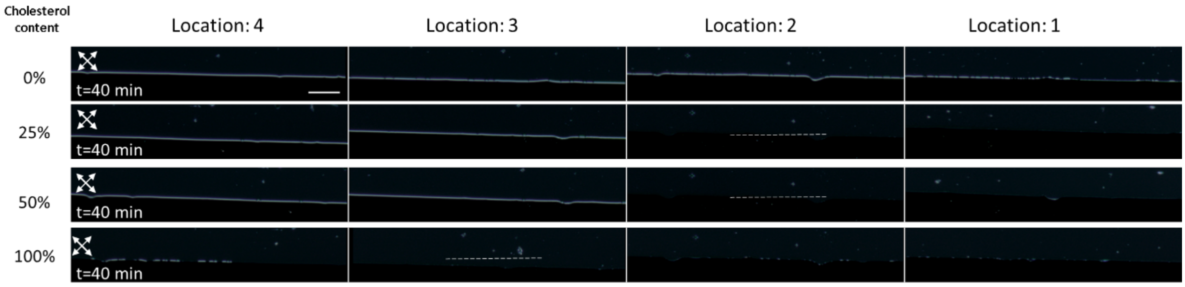

Figure S15. The polarized optical micrographs are shown that were taken from the 14 μm-deep microfluidic channels, where the aqueous phases were composed of 0.5 μM DPPC and varying cholesterol content from 0% to 75%. Every image was collected at t=40 min. The white double-sided arrow shows the orientations of the analyzer and polarizers used in all of the images shown in the figure. The dashed lines in the images indicate the location of the horizontal soft interfaces. Scale bar: 100 μm.

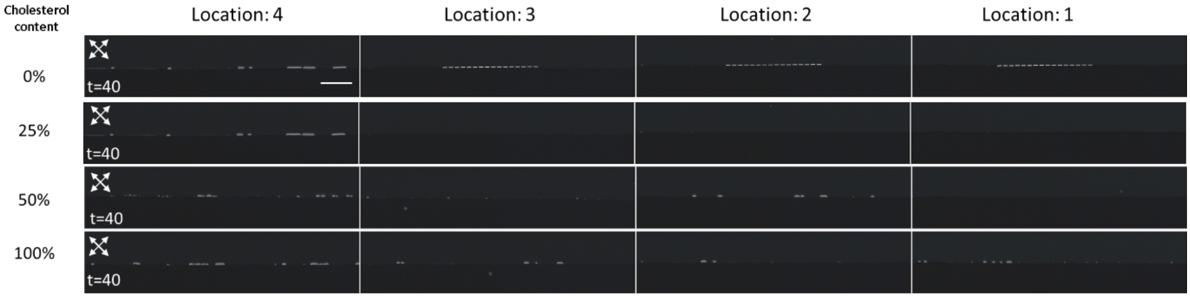

Figure S16. The polarized optical micrographs are shown that were taken from the 14 μm-deep microfluidic channels, where the aqueous phases were composed of 0.5 μM Egg SM and varying cholesterol content from 0% to 75%. Every image was collected at t=40 min. The white double-sided arrow shows the orientations of the analyzer and polarizers used in all of the images shown in the figure. The dashed lines in the images indicate the location of the horizontal soft interfaces. Scale bar: 100 μm.

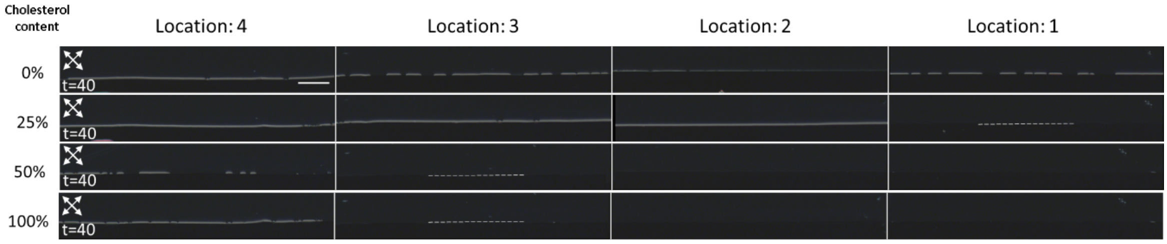

Figure S17. The polarized optical micrographs are shown that were taken from the 14 μm-deep microfluidic channels, where the aqueous phases were composed of 0.5 μM DOPC and varying cholesterol content from 0% to 75%. Every image was collected at t=40 min. The white double-sided arrow shows the orientations of the analyzer and polarizers used in all of the images shown in the figure. The dashed lines in the images indicate the location of the horizontal soft interfaces. Scale bar: 100 μm.

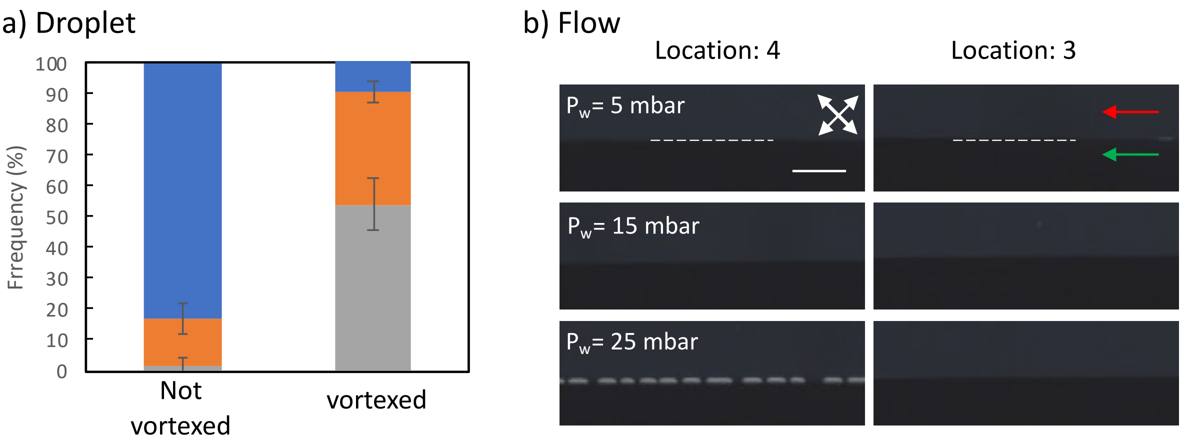

Figure S18. (a) Droplet configuration distributions for emulsions equilibrated with 50% cholesterol in 0.5 μM SM, as not vortexed and vortexed. (b) The polarized optical micrographs are shown that were taken from the 14 μm-deep microfluidic channels, where the aqueous phases were composed of 50% cholesterol in 0.5 μM SM. Images were collected at t=40 minutes, and at locations 4 and 3, as indicated in the top title. Images collected from the experiments flow were assisted by P_{LC} = 15 mbar: $P_{Aq.}$ = 5 mbar, $P_{Aq.}$ = 15 mbar, $P_{Aq.}$ = 25 mbar, as indicated in the images. The white double-sided arrow shows the orientations of the analyzer and polarizers used in all of the images shown in the figure. The dashed lines in the images indicate the location of the horizontal soft interfaces. Scale bar: 100 μm.

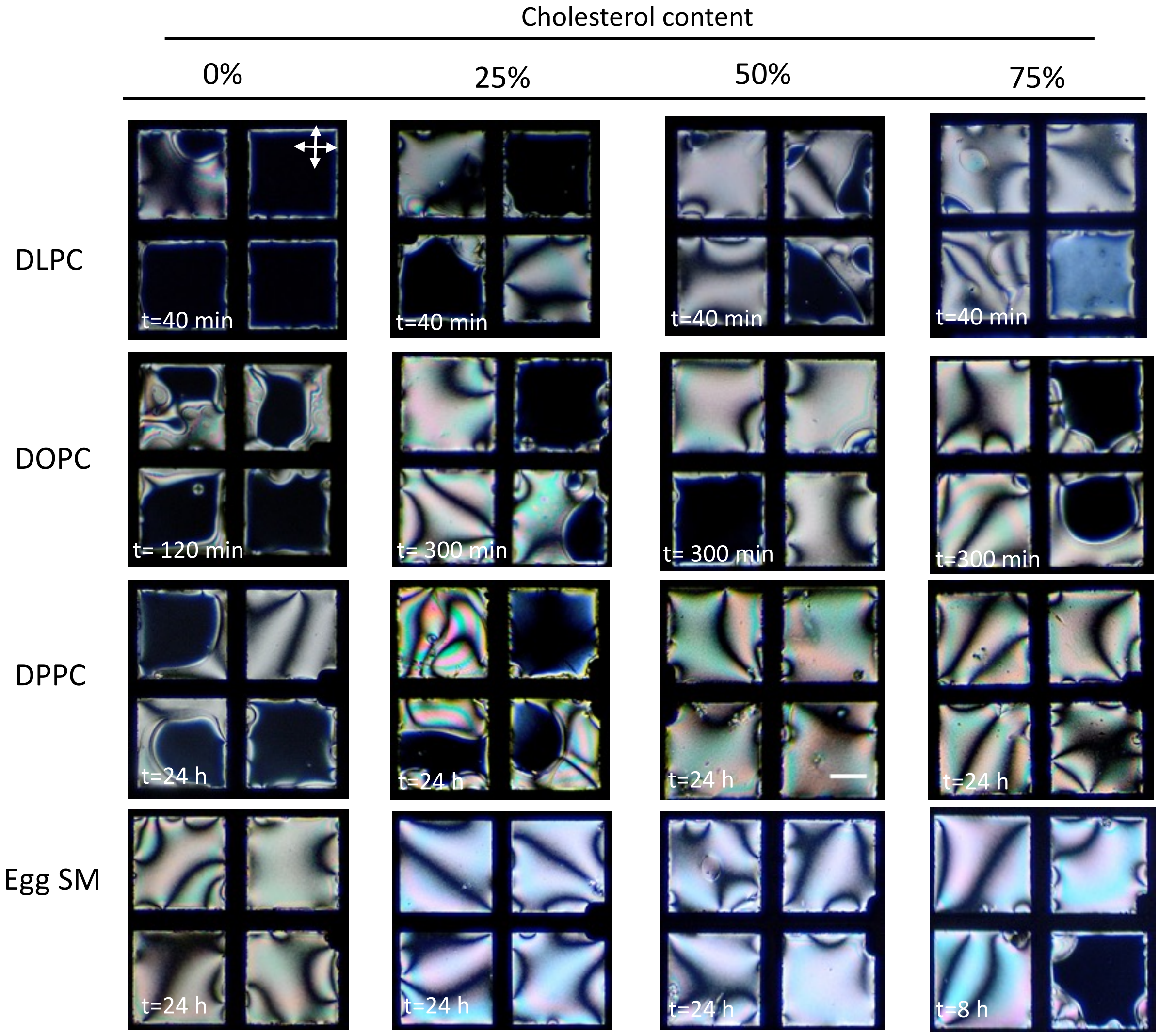

Figure S19. Polarized optical micrographs are collected under crossed polarizers and analyzer 0°- 90° for stagnant systems composed of an aqueous phase of 5.0 μM DLPC, DOPC, DPPC, and Egg SM, and varying cholesterol content from 0% to 75%. The white double-sided arrow shows the orientations of the analyzer and polarizers used in all of the images shown in the figure. Images collected during the duration after the aqueous phase was exchanged, as indicated in the images. Scale bar: 100

References

(1) Bruus, H. *Theoretical Microfluidics*, 3rd ed.; New York: Oxford University Press, 2006.

(2) Okiishi, M.; Rothmayer, H.; Huebsch, W.; Rothmayer, A. P. *Fluid Mechanics*, 7th ed.; John Wiley & Sons, 2013.

(3) Liu, Y.; Shen, L. From Langmuir Kinetics to First- and Second-Order Rate Equations for Adsorption. *Langmuir* **2008**, *24* (20), 11625–11630. https://doi.org/10.1021/la801839b.
